# Long-term outcomes and effectiveness of interventions in neonatal brachial plexus palsy: A systematic review

**DOI:** 10.1097/MD.0000000000044508

**Published:** 2025-09-12

**Authors:** José Alberto Pereira Pires, Italo C. Martins, Victor Arthur Ohannesian, Breno Dias L. Ribeiro, Bruno Marçola Ishizuka, Julia Sader Neves Ferreira, Matheus Almeida Cabral, Leonardo B.O. Brenner, Layza Hellen Fernandes Menezes, Almir Vieira Dibai Filho, Victor M. Lu, Rioko Kimiko Sakata, Ed Carlos Rey Moura, Plínio da Cunha Leal

**Affiliations:** a Department of Neurosurgery, Federal University of Maranhão, São Luís, MA, Brazil; b Department of Medicine, Federal University of Maranhão, São Luís, MA, Brazil; c School of Medicine, Albert Einstein Israeli Faculty of Health Sciences, São Paulo, Brazil; d Department of Medicine, Santo Amaro University, São Paulo, SP, Brazil; e Faculty of Medicine, Federal University of Goiás, Goiânia, GO, Brazil; f Department of Neurosurgery, State University of Ponta Grossa, Ponta Grossa, PR, Brazil; g Center for Sciences of Pinheiro (CCPi), Federal University of Maranhão, Pinheiro, MA, Brazil; h Department of Physical Education, Federal University of Maranhão, São Luís, MA, Brazil; i Department of Neurosurgery, University of Miami, FL; j Department of Anesthesiology, Pain, and Intensive Care Medicine, Escola Paulista de Medicina of Federal University of São Paulo (EPM/UNIFESP), SP, Brazil.

**Keywords:** functional recovery, long-term outcomes, NBPP, neonatal brachial plexus palsy, nerve reconstruction, systematic review

## Abstract

**Background:**

Neonatal brachial plexus palsy (NBPP) is a debilitating condition affecting approximately 1 in 1000 live births and often results in significant upper limb functional impairments. Despite advances in microsurgical techniques, long-term outcomes following various surgical interventions remain heterogeneous and controversial. This systematic review aimed to synthesize the evidence on the effectiveness of surgical treatments for NBPP, with a focus on long-term functional recovery across shoulder, elbow, hand, and wrist domains.

**Methods:**

Adhering to the Preferred Reporting Items for Systematic Reviews and Meta-Analyses 2020 guidelines, we conducted a comprehensive literature search across MEDLINE (via PubMed), Embase, Scopus, Web of Science, and the Cochrane CENTRAL Register of Controlled Trials. We included observational studies, cohort studies, and case series published from 1980 onward that reported long-term outcomes for NBPP.

**Results:**

A total of 965 patients from multiple countries were analyzed, with surgical interventions performed between 1990 and 2018. The mean age at surgery ranged from 0.2 to 7.9 years, with follow-up periods extending from 1 to over 10 years. Functional recovery varied by surgical approach, with nerve grafts and transfers showing significant improvements in upper limb mobility and strength. Shoulder function outcomes demonstrated gains in external rotation, abduction, and overall Mallet Score, while elbow and wrist functions showed enhanced active movement and strength. Hand functionality improved in select cases, though heterogeneity in assessment methods limited direct comparisons. Despite advances in surgical techniques, no single approach proved universally superior, highlighting the need for standardized outcome measures and high-quality prospective studies.

**Conclusion:**

Surgical reconstruction for obstetric brachial plexus palsy provides satisfactory functional recovery. However, outcome assessment remains challenging due to heterogeneous reporting, limiting a standardized evaluation of surgical efficacy. The predominance of retrospective studies and the lack of uniform clinical criteria further restrict the application of evidence-based medicine in this field. While nerve grafts and nerve transfers show promising results, there is no definitive consensus on their superiority, particularly regarding external shoulder rotation and hand function recovery.

## 1. Introduction

Neonatal brachial plexus palsy (NBPP) is a debilitating condition that primarily occurs during the perinatal period, characterized by paralysis, weakness, and loss of sensation in the affected limb.^[1]^ Various factors, including maternal conditions, newborn characteristics, and obstetric interventions, can lead to excessive forces being applied to the brachial plexus, a structure anatomically vulnerable to injury during childbirth.^[2]^ The incidence of NBPP is slightly over 1 in 1000 live births.^[3]^ The extent of the injury can vary from neurapraxia, which generally recovers without intervention, to complete avulsion, which lacks the capacity for spontaneous regeneration.^[4]^

Children affected by NBPP and their families face significant challenges, as the condition often results in economic burdens and reduced quality of life, particularly in cases associated with shoulder dystocia.^[5,6]^ Selecting candidates for surgery remains a complex issue, but early identification of neurotmetic lesions or nerve root avulsions is essential to minimize the impact of prolonged denervation.^[7]^ Newborns with NBPP generally have a favorable prognosis; however, early referral to specialized clinics is critical to optimizing care and leveraging advances in treatment.^[8]^

The success of brachial plexus surgery depends on a thorough understanding of the intricate nerve connections and adjacent structures.^[9]^ The primary goal is to restore hand grasp, followed by elbow flexion, shoulder mobility, and extension of the elbow, wrist, and fingers. To restore lost motor and sensory functions, both extraplexal and intraplexal nerve transfers are commonly performed.^[10]^

Despite significant advancements in brachial plexus surgery, there is still a lack of comprehensive and systematic studies on the indications, techniques, and outcomes of these procedures. Additionally, the current literature insufficiently addresses the effectiveness and long-term results of surgical interventions. Furthermore, the implementation of evidence-based medicine in the treatment of obstetric brachial plexus palsy (OBPL) presents considerable challenges, which further complicates the establishment of standardized guidelines. Thus, the aim of this study is to perform a systematic review of the literature to assess the success rates of surgical treatments for brachial plexus injuries, with a particular focus on the long-term follow-up of patients, taking into account different types of injuries and various surgical techniques. Based on the findings, a discussion will be developed grounded in the available evidence.

## 2. Methods

This systematic review was conducted following the Preferred Reporting Items for Systematic Reviews and Meta-Analyses (2020) guidelines.^[11]^ The protocol was prospectively registered in the International Prospective Register of Systematic Reviews (registration number: CRD42025639917).

### 2.1. Literature search

A comprehensive literature search was performed across 5 databases: MEDLINE (via PubMed), Embase, Scopus, Web of Science, and the Cochrane CENTRAL Register of Controlled Trials. The search strategy combined terms related to neonatal brachial plexus injury (e.g., “brachial plexus injury,” “Erb palsy,” “obstetric brachial plexus”) with terms referring to long-term outcomes (e.g., “functional outcomes,” “prognosis,” “motor recovery,” “quality of life”) and pediatric populations (e.g., “newborn,” “infants,” “children”). The search strategy was developed and peer-reviewed according to the Peer Review of Electronic Search Strategies 2015 Guideline Statement.^[12]^ No restrictions on language or publication date were applied. Additionally, reference lists of all included articles were examined through a snowballing approach to identify further relevant studies. Full search strategies for each database are provided in the Table S1, Supplemental Digital Content, https://links.lww.com/MD/P937.

### 2.2. Eligibility criteria

The initial search, conducted in September 2024, identified 1866 articles. After duplicate removal, 1423 studies remained for title and abstract screening. Screening and selection were conducted independently by 2 reviewers (V.A.O. and J.S.N.F.), with disagreements resolved by a third reviewer (I.C.M.). Eligibility was determined based on the PICOTT framework: Population (P): newborns, infants, and children diagnosed with NBPP. Intervention (I): surgical or non-surgical interventions with reported long-term outcomes. Comparator (C): not pre-specified; studies with diverse management strategies or follow-up durations were considered. Outcomes (O): long-term functional recovery (defined as a minimum follow-up period of 3 years), motor recovery, prognosis, and quality of life. Time (T): studies published from 1980 onwards. Type of studies (T): observational studies, cohort studies, and case series. Studies were excluded if they were case reports or did not report sufficient data on long-term outcomes.

### 2.3. Approach to data heterogeneity

Given the substantial clinical and methodological heterogeneity (including variations in intervention types [e.g., nerve surgeries vs musculoskeletal procedures], outcome measures, and follow-up periods) the review was explicitly designed as a descriptive synthesis. No attempts were made to compare distinct categories of interventions directly (e.g., nerve surgeries vs muscle/tendon surgeries), recognizing their differing indications, timing, and mechanisms of action.

### 2.4. Quality assessment

Two reviewers (M.C.A. and V.A.O.) independently assessed the risk of bias of each included study using the Risk of Bias in Non-Randomized Studies – of Interventions, tool.^[13]^ This evaluation considered domains such as confounding, selection bias, classification of interventions, missing data, outcome measurement, and reporting bias. Studies were categorized as having low, moderate, serious, or critical risk of bias. Any discrepancies were resolved by consensus between the reviewers.

### 2.5. Data extraction and synthesis

Key data, including participant characteristics, details of interventions, and long-term outcomes, were extracted. Given the heterogeneity across studies, the data were organized according to thematic domains, specifically: functional recovery, motor recovery, prognosis, and quality of life. In alignment with the nature of the available evidence, no quantitative or statistical synthesis (e.g., meta-analysis) was conducted. Subgroup and sensitivity analyses were not feasible due to the variability in interventions, populations, and outcome measures. All findings were presented descriptively in tables and narrative summaries.

This approach was intentionally chosen to respect the heterogeneity and avoid inappropriate comparisons, particularly between interventions of distinct clinical purposes, such as nerve reconstruction and musculoskeletal surgeries.

### 2.6. Ethical considerations

As this review exclusively analyzed publicly available data from published studies, ethical approval was not required. No individual-level, sensitive, or identifiable data were collected. The study adhered to all ethical guidelines for conducting research based on published literature, ensuring the integrity and confidentiality of the data analyzed.

## 3. Results

### 3.1. Study selection

**Figure 1.**
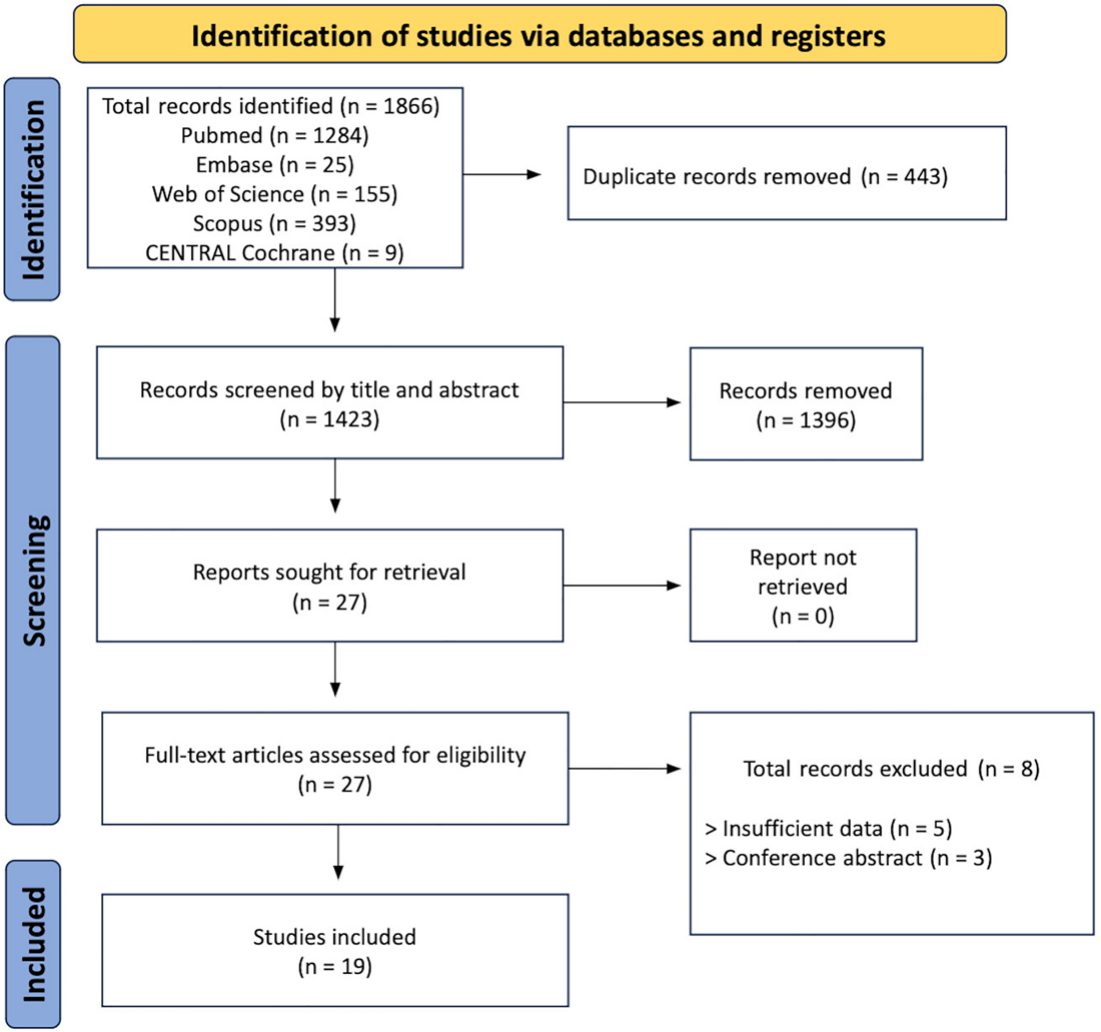
PRISMA flowchart illustrating the search strategy. PRISMA = Preferred Reporting Items for Systematic Reviews and Meta-Analyses.

A total of 1866 articles were retrieved from PubMed (MEDLINE), Embase, Scopus, Web of Science, and CENTRAL Cochrane. After excluding 443 duplicates, 1423 records underwent title and abstract screening, resulting in the exclusion of 1396 articles for not meeting the inclusion criteria. Twenty-seven studies were selected for full-text assessment, of which 5 were excluded due to insufficient data and 3 were excluded for being conference abstracts. Ultimately, 19 studies met the eligibility criteria and were included in the systematic review. The Preferred Reporting Items for Systematic Reviews and Meta-Analyses flow diagram (Fig. 1) provides a detailed overview of the selection process.

### 3.2. Baseline characteristics of included studies

A total of 965 patients were included, with data collected between 1990 and 2018 across multiple countries, including the USA, UK, France, Egypt, Brazil, Austria, Norway, Sweden, Finland, Canada, and the Netherlands. The population consisted of both male and female participants, with a gender distribution ranging from 71/29 to 28/72%. The mean age at the time of surgery varied from 0.2 to 7.9 years, and follow-up durations ranged from 1 to over 10 years.

Injury severity was divided into different grades (G1–G4), according to the Narakas Classification,^[14]^ with G1 being the most frequent. Surgical interventions varied according to injury severity and clinical presentation, including nerve reconstruction, grafting, neurolysis, tendon and muscle transfers, arthroscopy, and contracture release. Procedures such as the Mod Quad surgery, Triangle Tilt, and Oberlin technique were reported in selected cases. The variability in surgical approaches reflects the heterogeneity of treatment strategies across different centers.

The mean follow-up period ranged from 4 to 13.3 years, allowing for the assessment of long-term outcomes. Treatment efficacy was evaluated based on clinical and functional recovery, with variations in assessment methods across studies. The heterogeneity in populations and surgical techniques provides a comprehensive perspective on the management of NBPP, contributing to a broader understanding of patient outcomes. Information on each study is available in Table 1.

**Table 1:** Characteristics of included studies.

Study				Population					Procedure
Authors	Country	Type	Data collection Period	N	M/F	Type of injury n (%)	Age at surgery Mean (range), yr	Follow-up Mean (range), yr	Surgery intervention
Aber et al^[15]^	USA	R	2000–2005	32	13/19	G1: 32 (100)	0.8 ± 0.37 (N/A)	15 ± 2.17 (12.8–17.2)	Nerve reconstruction
Anand & Birch^[16]^	UK	R	N/A	24	N/A	N/A	N/A	N/A (3–23)	Nerve grafts, reconstruction and transfer
Bisinella et al^[17]^	UK	P	1994–1996	73	33/40	G1: 20 (27) G2: 45 (62) G3: 8 (11)	N/A	4 ± N/A (3–6)	N/A
Breton et al^[18]^	France	R	2004–2010	18	5/13	G1: 9 (50) G2: 4 (22) G3 or G4: 5 (28)	4.2 ± N/A (1–11)	4.5 ± N/A (1–7)	Arthroscopy
Butler et al^[19]^	USA	R	2007–2012	56	20/36	G1: 21 (38) G2: 8 (14) G3: 17 (30) G4: 10 (18)	N/A	N/A (16–28)	N/A
El-gammal et al^[20]^	Egypt	P	N/A	26	17/9	G1 and G2	3 ± N/A 1–10	8.2 ± 3.83 (5–16)	Teres major to infraspinatus transfer
El-gammal et al^[21]^	Egypt	R	2000–2014	29	19/10	G1: 5 (17) G2: 4 (14) G3: 20 (69)	1.3 ± N/A (1–3.5)	7.9 ± N/A (2.3–16)	Neurolysis, grafting and neurotization
Figueiredo et al^[22]^	Brazil	P	2010–2018	11	N/A	G1: 2 (18) G2: 5 (46) G3: 4 (36)	7.9 ± 2.7 (5–12)	11.2 ± 5.3 (3–19.8)	Oberlin technique and neurotization
Gmeiner et al^[23]^	Austria	R	2000–2007	14	10/4	N/A	3.7 ± 2.8 (1.4–10.9)	8.5 ± 2.5 (N/A)	Nerve transfer and reconstruction
Hulleberg et al^[24]^	Norway	R	1991–2000	69	36/33	G1, G2 and G3	N/A	14 (10–20) [Median]	Sural nerve grafting and secondary reconstruction
Hultgren et al^[25]^	Sweden	R	1997–2009	118	54/64	G1 or G2: 116 (98) G3: 2 (2)	N/A	10.4 ± N/A (7–15.1)	Subscapularis tendon division and reparation
Jonsson et al^[26]^	Sweden	R	1997–2009	61	21/40	G1: 59 (97) G3: 2 (3)	3.2 ± N/A (0.7–15)	10.2 ± N/A (7–16)	Elongation of the subscapularis tendon
Kirjavainen et al^[27]^	Finland	R	2002–2003	112	47/65	G1: 54 (48) G2: 31 (28) G3 or G4: 27 (24)	0.2 ± N/A (0.03–1.1)	13.3 ± N/A (5–31.5)	Neurolysis, neurorrhaphy, grafting, neurotization
Manske et al^[28]^	USA	R	1990–2014	43	26/17	G1: 16 (37) G2: 11 (26) G3: 3 (7) G4: 13 (30)	7.1 ± N/A (3.5–12)	7.4 (2–18.1)	Reconstruction with sural nerve autograft
Morrow et al^[29]^	Canada	R	1992–2016	50	26/24	N/A	N/A	N/A (4–8)	Brachial plexus reconstruction
Nath & Liu^[30]^	USA	R	2005–2008	75	38/37	N/A	5.8 ± N/A (2.1–11.8)	7 (2.7–13.3)	Triangle tilt surgery, mod quad procedure, nerve grafting and transfer
Nath & Somasundaram^[31]^	USA	R	2005–2008	22	13/9	G1: 11 G2: 7 G3: 4	5.8 ± N/A (2.1–11.8)	5	Mod quad and triangle tilt surgeries
Nath & Somasundaram^[32]^	USA	R	2005–2018	17	8/9	G1: 7 G2: 8 G3: 1 G4: 1	1.94 ± N/A (0.5–6.5)	Minimum of 10	Mod quad and triangle tilt surgeries
Van der Holst et al^[33]^	Netherlands	R	2013–2014	115	60/55	G1: 68 G2: 40 G3: 4 G4: 3	4.7 ± 3.3 (N/A)	6 ± 3.2 (N/A)	Internal contracture release and/or muscle tendon transfer

G1 = Narakas Group 1 (C5–C6 injury), G2 = Narakas Group 2 (C5–C7 injury), G3 = Narakas Group 3 (C5–T1 injury with no Horner), G4 = Narakas Group 4 (C5–T1 injury with Horner), N/A = not available, P = prospective study, R = retrospective study, USA = United States of America, UK = United Kingdom.

### 3.3. Quality assessment of the included studies

**Figure 2.**
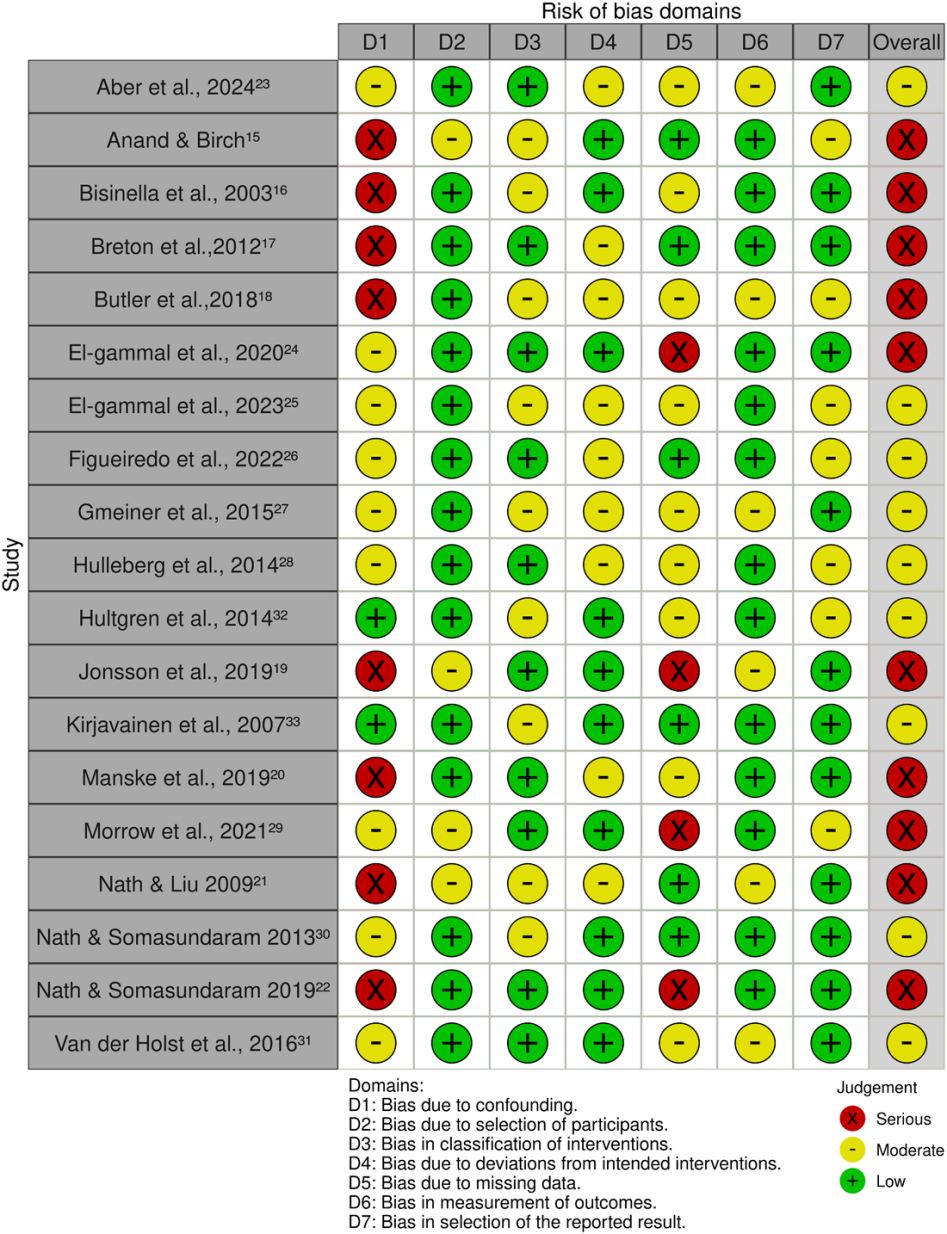
Traffic light plot.

**Figure 3.**
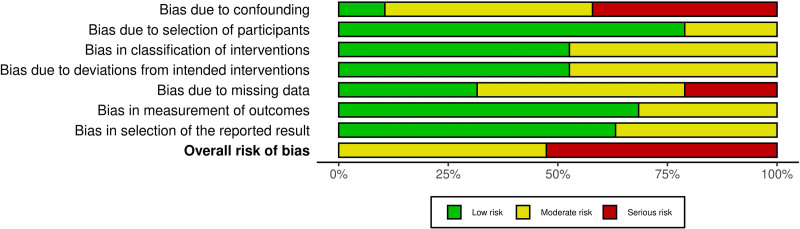
Summary plot.

The results of the risk of bias assessment, using the Risk of Bias in Non-Randomized Studies – of Interventions, Version 2, are presented in the traffic light plot (Fig. 2), which shows the risk of bias for each domain, and in the summary plot (Fig. 3), which illustrates the overall distribution of risk.

In Domain 1 (bias due to confounding), 8 of the 19 studies were classified as having a serious risk of bias, due to inadequate control of confounding variables and the absence of appropriate statistical models to address these factors.^[16–19,26,28,30,32]^ Conversely, 9 studies were categorized as having a moderate risk, as confounding factors were not clearly addressed, although additional analyses did not indicate relevant unmeasured confounding.^[15,20–24,29,31,33]^ Two studies were considered to have a low risk of bias, demonstrating either adequate control of potential confounders or minimal impact of such factors.^[25,27]^

In Domain 2 (bias in the selection of participants), 4 studies presented a moderate risk of bias, generally due to retrospective selection without standardized post-intervention inclusion criteria.^[16,26,29,30]^ The remaining studies were classified as low risk, as they presented clear and traceable inclusion criteria.^[15,17–25,27,28,31–33]^

In Domain 3 (bias in the classification of interventions), 10 studies presented a low risk of bias, since the interventions (mainly surgical) were clearly described, standardized, and applied uniformly.^[15,18,20,22,24,25,28,29,32,33]^ Nine studies were classified as having a moderate risk in this domain, due to retrospective classification and the potential for measurement bias from prior knowledge of outcomes.^[16,17,19,21,23,26,27,30,31]^

In Domain 4 (bias due to deviations from intended interventions), 9 studies were classified as having a moderate risk, due to minor variations in procedures, absence of explicit protocols, or lack of statistical adjustment for such deviations.^[15,18,19,21–24,28,30]^ The remaining studies, which showed good standardization and consistency in intervention implementation, were assessed as low risk.^[16,17,20,25–27,29,31–33]^

In Domain 5 (bias due to missing data), 4 studies presented a serious risk, with substantial data loss, particularly related to confounding variables, without the use of appropriate methods to handle these losses.^[20,26,29,30]^ Nine studies exhibited a moderate risk, due to follow-up losses and lack of clear information on how missing data were managed.^[15,17,19,21,23–25,28,33]^ Only 6 studies were assessed as having a low risk in this domain, for presenting adequate follow-up and complete patient evaluation.^[16,18,22,27,31,32]^

In Domain 6 (bias in the measurement of outcomes), the majority of studies (13 out of 19) were classified as having a low risk, as they used objective assessment methods or applied partial blinding.^[16–18,20–22,24,25,27–29,31,32]^ Only 6 studies were considered to be at moderate risk, as they used subjective outcomes without reporting assessor blinding, thereby increasing the likelihood of bias.^[15,19,23,26,30,33]^

Finally, in Domain 7 (bias in the selection of the reported results), 7 studies were classified as having a moderate risk, mainly due to the absence of a pre-registered protocol or incomplete reporting of subgroups and planned analyses.^[16,19,21,22,24,25,29]^ The other twelve studies were assessed as low risk, as they presented complete and consistent reporting of expected outcomes.^[15,17,18,20,23,26–28,30–33]^

According to Cochrane guidelines, 10 studies were classified as having an overall high risk of bias,^[16–20,26,28–30,32]^ while 9 presented a moderate risk.^[15,21–25,27,31,33]^

### 3.4. Outcomes of included studies

This systematic review investigated the long-term functional outcomes of NBPP by evaluating specific functional domains: shoulder function, elbow function, hand function, wrist function, and overall upper limb functionality. Additionally, key functional metrics such as the Mallet Score and Disabilities of the Arm, Shoulder and Hand Score were highlighted.

Shoulder recovery was the most frequently reported outcome across studies and improvements were consistently documented in external rotation, abduction, and global shoulder performance. Bisinella et al reported 94% and 96% success rates in active shoulder rotation recovery for non-operated C5 and C6 injuries, respectively.^[17]^ Arthroscopic procedures also showed benefits, with Breton et al reporting improvements of up to 18° in active and 30% in passive external rotation.^[18]^ Using the Mallet Score, Nath et al documented notable increases across multiple shoulder movements, including external rotation, hand-to-neck, and hand-to-spine motions, with the total Mallet Score improving from 14.1 ± 2.7 to 20.3 ± 2.5.^[31]^ Similarly, Manske et al reported sustained recovery of abduction and external rotation (Active Movement Scale [AMS] > 5) following sural nerve grafting, with follow-up durations ranging from 18 to 30 months.^[28]^ Procedures combining nerve and musculoskeletal components also yielded substantial results. Hultgren et al observed increases of 103.6° in abduction and 22.6° in external rotation after subscapularis elongation combined with nerve transfer.^[25]^ In cases of total NBPP (C5–T1), Morrow et al reported modest gains of 1.7 ± 1.4 points in abduction and 0.4 ± 0.4 in external rotation on the AMS.^[29]^ El-Gammal et al documented abduction improvements of up to 109.6° ± 27.2° following Teres Major muscle transfer.^[21]^

Elbow function outcomes were favorable in the studies that addressed this domain, particularly in terms of flexion recovery. Interventions involving neurolysis and nerve grafting demonstrated substantial functional gains, with Toronto Active Movement Scale (TAMS) scores exceeding 6 in the majority of patients.^[21]^ Similarly, Morrow et al observed average elbow flexion scores of 5.2 ± 1.0 on the AMS following primary nerve reconstruction.^[29]^

The recovery of hand function showed more variable success. Following nerve grafting, 38% of patients achieved satisfactory hand function (Raimondi > 3).^[28]^ In Nath et al, hand functionality measured by the Brachial Plexus Outcome Measure (BPOM) showed significant improvements, with scores reaching 29 points 4 years post-surgery.^[32]^

Targeted interventions also proved effective in restoring wrist mobility and strength. Success rates for wrist function recovery, measured by the BPOM Subscale, were high, reaching 83% (24 out of 29) at the 4-year follow-up and 81% (21 out of 26) at the 8-year follow-up.^[29]^ After sural nerve autografting, 37% of patients achieved functional wrist extension (AMS > 5).^[28]^ In 1 study, a 100% success rate in achieving TAMS > 6 for wrist flexion and extension was reported; however, it is important to note that this specific finding was based on the evaluation of a single patient.^[21]^

Beyond specific joint movements, the studies demonstrated that surgical interventions had a positive impact on overall upper limb function, quality of life, and patient satisfaction. Global functionality scores and quality of life improved in both early and delayed intervention groups.^[26]^ This was quantitatively supported by significant improvements in postoperative Disabilities of the Arm, Shoulder and Hand scores, indicating enhanced function and reduced disability.^[22]^ Strength recovery was also notable, with mean strength improving to 4.09 ± 1.45 on the Medical Research Council scale in 1 study.^[22]^ Finally, surgical intervention also addressed cosmetic aspects, such as correcting glenoid retroversion by 25.4°,^[27]^ and achieved high patient satisfaction rates, reaching 79% in those undergoing combined surgical approaches.^[25]^

## 4. Discussion

This study evaluated the outcomes of surgical interventions for OBPL. The diversity of surgical techniques reported in the literature reflects the condition’s complexity and the need for tailored approaches.^[7–9]^ While many of the included studies were retrospective, their collective data provide a substantial evidence base. This discussion synthesizes these findings, contextualizes them within the existing literature, and addresses the key limitations observed, such as the heterogeneity of clinical outcomes and the lack of uniform assessment protocols.

The findings of this systematic review indicate a clinically relevant functional recovery of the shoulder joint after nerve reconstruction, evidenced by a significant improvement in the aggregated Mallet Score from 14.1 ± 2.7 to 20.3 ± 2.5.^[31]^ These results align with previous studies, such as Manske et al, who reported consistent recovery in abduction and external rotation (AMS > 5) with sural nerve grafting over a mean follow-up of 7.4 years.^[28]^ Furthermore, our review supports the effectiveness of combined procedures. Hultgren et al demonstrated that subscapular elongation with nerve transfer resulted in a 103.6° increase in shoulder abduction and a 22.6° improvement in external rotation.^[25]^ It is noteworthy that such combined procedures involve both musculoskeletal and nerve interventions, making direct comparisons with other surgical strategies methodologically challenging.

In cases of total OBPL (C5–T1), the observed recovery was more modest, with an average increase of 1.7 ± 1.4 points in shoulder abduction and 0.4 ± 0.4 points in external rotation on the AMS.^[29]^ These findings reflect the continuous progress in nerve reconstruction techniques.^[34]^ However, the debate over the superiority of nerve grafts versus nerve transfers persists. Some studies suggest a slight advantage for nerve transfers in recovering external rotation,^[35]^ potentially because they allow for faster reinnervation by being performed closer to the target muscle.^[36]^ Nonetheless, due to significant heterogeneity across studies and a lack of high-quality comparative trials, definitive conclusions cannot be drawn.

Regarding elbow recovery, this review found that nerve reconstruction showed favorable outcomes. El-Gammal et al reported significant gains in elbow flexion and extension strength after neurolysis and grafting, with most patients achieving TAMS scores exceeding 6.^[21]^ Similarly, Morrow et al observed mean AMS elbow flexion scores of 5.2 ± 1.0 after primary nerve reconstruction.^[29]^ These outcomes are consistent with a meta-analysis by Tora et al, which found that patients achieved functional elbow flexion with no substantial differences between nerve grafting and nerve transfer techniques.^[37]^ Thus, both strategies appear to yield comparable outcomes for elbow function.

Wrist function also demonstrated substantial recovery following targeted interventions. El-Gammal et al reported TAMS > 6 for wrist flexion and extension (although this finding was based on a single case) following neurotization procedures,^[21]^ while Manske et al observed that 37% of patients achieved functional wrist extension (AMS > 5) after sural nerve autografting.^[28]^ Morrow et al documented success rates for wrist function recovery, as measured by the BPOM Wrist, Finger, and Thumb Subscale, of 83% at 4 years and 81% at 8 years.^[29]^ Nonetheless, variability in surgical techniques and patient characteristics across studies complicates the generalization of these outcomes. These findings support the observation that functional gains can continue for 3 or more years after surgery, highlighting the importance of long-term follow-up.^[38]^

In contrast, the recovery of hand function remains a significant challenge. Although impaired hand function is an absolute indication for early surgery,^[38,39]^ outcomes are variable. Manske et al reported that only 38% of patients achieved satisfactory hand function (Raimondi > 3) after nerve grafting.^[28]^ While Nath et al documented significant improvements on the BPOM,^[32]^ overall recovery of hand function is less predictable than that of more proximal joints, likely due to the severity of the initial injury and individual differences in neural plasticity.

In summary, this review confirms that surgical intervention plays a fundamental role in the functional recovery of patients with obstetric brachial plexus injuries. Surgery is particularly effective in restoring elbow flexion and shoulder stability. While early intervention is often advocated for severe injuries, the optimal timing remains dependent on the type of lesion and potential for spontaneous recovery. Therefore, surgical indications must be based on a careful, individualized analysis of a patient’s functional prognosis against the risk-benefit ratio. To move forward, future research must prioritize standardizing outcome measures and conducting prospective comparative studies to reduce the evidence heterogeneity that currently limits the field.

## 5. Limitations

The main limitation of this study relates to the predominance of retrospective designs in the available literature on brachial plexus palsy, which inherently reduces the strength and reliability of the evidence base. Furthermore, substantial heterogeneity was observed in outcome reporting, as different studies employed diverse functional assessment scales and varied criteria for evaluating surgical success, thereby hindering direct comparisons between interventions. The lack of access to individual patient-level data in several studies also precluded more granular analyses of prognostic factors that could influence surgical outcomes. Additionally, the inclusion of studies conducted across different geographic regions and over extended time periods may have introduced variability, particularly given the evolution of surgical techniques and rehabilitation protocols. Finally, the persistent absence of robust, prospective, and well-designed studies with larger, standardized cohorts represents a significant gap, underscoring the need for future research to establish clearer evidence-based guidelines for the management of brachial plexus palsy.

## 6. Conclusion

Surgical interventions for OBPL demonstrably improve functional outcomes, particularly for shoulder and elbow mobility. However, the evidence base is fundamentally limited by the predominance of retrospective studies with a high risk of bias and significant heterogeneity in techniques, follow-up times, and outcome measures. This prevents a standardized evaluation of efficacy and precludes definitive conclusions on the superiority of any single technique. To advance the field and enable true evidence-based practice, a clear roadmap is necessary: future research must prioritize the development of standardized outcome reporting and the execution of prospective, comparative studies. Only through the generation of robust, high-quality evidence can clinical practice be truly optimized for these patients.

## Author contributions

**Conceptualization:** Italo C. Martins, Julia Sader Neves Ferreira, Layza Hellen Fernandes Menezes.

**Data curation:** Breno Dias L. Ribeiro, Bruno Marçola Ishizuka, Julia Sader Neves Ferreira.

**Investigation:** Julia Sader Neves Ferreira, Leonardo B. O. Brenner, Ed Carlos Rey Moura, Almir Vieira Dibai Filho.

**Methodology:** José Alberto Pereira Pires, Victor Arthur Ohannesian, Matheus Almeida Cabral, Leonardo B. O. Brenner.

**Project administration:** José Alberto Pereira Pires, Italo C. Martins, Ed Carlos Rey Moura, Plínio da Cunha Leal.

**Supervision:** José Alberto Pereira Pires, Victor M. Lu, Rioko Kimiko Sakata, Ed Carlos Rey Moura, Plínio da Cunha Leal.

**Software:** Victor Arthur Ohannesian, Bruno Marçola Ishizuka, Matheus Almeida Cabral.

**Validation:** Italo C. Martins.

**Visualization:** Victor Arthur Ohannesian, Matheus Almeida Cabral, Almir Vieira Dibai Filho.

**Writing – original draft:** José Alberto Pereira Pires, Italo C. Martins, Breno Dias L. Ribeiro, Bruno Marçola Ishizuka, Matheus Almeida Cabral, Layza Hellen Fernandes Menezes, Almir Vieira Dibai Filho.

**Writing – review & editing:** José Alberto Pereira Pires, Italo C. Martins, Victor Arthur Ohannesian, Breno Dias L. Ribeiro, Julia Sader Neves Ferreira, Leonardo B. O. Brenner, Layza Hellen Fernandes Menezes, Victor M. Lu, Rioko Kimiko Sakata, Ed Carlos Rey Moura, Almir Vieira Dibai Filho, Plínio da Cunha Leal.

## Supplementary Material


