# Supplementary material for: Long-term outcomes and effectiveness of interventions in neonatal brachial plexus palsy: A systematic review
Source: Medicine (Baltimore). 2025 Sep 12;104(37):e44508. doi: 10.1097/MD.0000000000044508 (PMC12440485; doi:10.1097/MD.0000000000044508)
Supplement: Supplementary file 1 [file medi-104-e44508-s001.docx]

**S1. Search Strategy**

| **Database** | **N** | **Search** |
| --- | --- | --- |
| **MEDLINE**  **(PubMed)** | 1284 | (brachial plexus injury OR brachial plexus palsy OR "Erb's palsy" OR "Klumpke's palsy" OR obstetric brachial plexus) AND ("long-term follow-up" OR functional outcomes OR prognosis OR motor recovery OR "quality of life") AND  (newborn OR infants OR children) |
| **Embase** | **25** | ('brachial plexus injury'/exp OR 'brachial plexus injury':ti,ab,kw,de,dn,df,mn,tn OR 'brachial plexus palsy'/exp OR 'brachial plexus palsy':ti,ab,kw,de,dn,df,mn,tn OR 'erb palsy'/exp OR 'erb palsy':ti,ab,kw,de,dn,df,mn,tn OR 'klumpke palsy'/exp OR 'klumpke palsy':ti,ab,kw,de,dn,df,mn,tn OR 'obstetric brachial plexus injury'/exp OR 'obstetric brachial plexus injury':ti,ab,kw,de,dn,df,mn,tn) AND ('long term follow up'/exp OR 'long term follow up':ti,ab,kw,de,dn,df,mn,tn OR 'functional outcomes'/exp OR 'functional outcomes':ti,ab,kw,de,dn,df,mn,tn OR prognosis/exp OR prognosis:ti,ab,kw,de,dn,df,mn,tn OR 'motor recovery'/exp OR 'motor recovery':ti,ab,kw,de,dn,df,mn,tn OR 'quality of life'/exp OR 'quality of life':ti,ab,kw,de,dn,df,mn,tn) AND (newborn/exp OR newborn:ti,ab,kw,de,dn,df,mn,tn OR infant/exp OR infant:ti,ab,kw,de,dn,df,mn,tn OR child/exp OR child:ti,ab,kw,de,dn,df,mn,tn) |
| **Web Of Science** | **155** | TS=("brachial plexus injury" OR "brachial plexus palsy" OR "Erb's palsy" OR "Klumpke's palsy" OR "obstetric brachial plexus") AND TS=("long-term follow-up" OR "functional outcomes" OR prognosis OR "motor recovery" OR "quality of life") AND TS=(newborn OR infants OR children) |
| **Scopus** | **393** | (TITLE-ABS-KEY("brachial plexus injury" OR "brachial plexus palsy" OR "Erb's palsy" OR "Klumpke's palsy" OR "obstetric brachial plexus")) AND (TITLE-ABS-KEY("long-term follow-up" OR "functional outcomes" OR prognosis OR "motor recovery" OR "quality of life")) AND (TITLE-ABS-KEY(newborn OR infants OR children)) |
| **Cochrane (CENTRAL)** | **9** | ("Brachial Plexus Injury" OR "Brachial Plexus Palsy" OR "Erb's Palsy" OR "Klumpke's Palsy" OR "Obstetric Brachial Plexus") AND ("Long-Term Follow-Up" OR "Functional Outcomes" OR "Prognosis" OR "Motor Recovery" OR "Quality of Life") AND ("Newborn" OR "Infants" OR "Children") |
| **Total** | **1866** | |
